# *In vitro* Analysis of DSPP and BSP Expression: Comparing the Odontogenic Influence of Bio-C Repair and Biodentine in hDPSCs

**DOI:** 10.1055/s-0044-1786984

**Published:** 2024-07-19

**Authors:** Valeria Widita Wairooy, Dini Asrianti Bagio, Anggraini Margono, Ingrid Amelia

**Affiliations:** 1 Department of Conservative Dentistry, Faculty of Dentistry, Universitas Indonesia, Jakarta, Indonesia

**Keywords:** bone sialoprotein, calcium silicate, dental pulp, dentin sialophosphoprotein, stem cells

## Abstract

**Objectives** This study compared the ability of BIO-C Repair (BC) and Biodentine (BD) in relation to odontogenic differentiation by evaluating the dentin sialophosphoprotein (DSPP) and bone sialoprotein (BSP) expression and mineral deposition of human dental pulp stem cells (hDPSCs).

**Materials and Methods** BC and BD were pulverized and sterilized (ISO 10993-5:2009). The hDPSCs were the result of primary cultures that were 80% confluent (having gone through the stem cell marker tests CD90 98%, CD105 99.7%, CD73 94%, and LinNeg 0.5%) and reached P2–3 by means of serum starvation for 24 hours. This study involved seven groups, in which the hDPSCs were cultured on osteogenic media with the addition of either BD (Septodont, United States) at concentrations of 1:1, 1:2, or 1:5; BC (Angelus, Brazil) at concentrations of 1:1, 1:2, or 1:5; or the negative control (Dulbecco's modified eagle medium + osteogenic media). The hDPSC differentiation was determined via enzyme-linked immunosorbent assays of DSPP and BSP expression performed on days 7 and 14 and alizarin red staining performed on day 21.

**Statistical Analysis** The data were analyzed using a one-way analysis of variance, followed by Tamhane's post hoc test, to compare the differences between groups. The *t* -test dependent was also used to identify differences between groups.

**Results** BC and BD at 1:1 concentration, there was a statistically significant difference in DSPP and BSP expression. However, at concentrations of 1:2 and 1:5, there was no significant difference observed in either duration of observation ( *p* > 0.05). The highest DSPP and BSP concentrations after 7 and 14 days of observation were observed with BD and BC at 1:5 concentration (6.6–6.71 and 13.20–13.47 ng/mL).

**Conclusion** The study shows that BC is as effective as BD in enhancing DSPP and BSP expression and mineral deposition in hDPSCs. The 1:5 concentration of BC showed the highest levels of DSPP and BSP expression and mineral deposition.

## Introduction

Calcium silicate cement is a scaffold that can stimulate the regeneration of the dentin–pulp complex. 1 Moreover, the development of calcium silicate cement has changed the chemical composition and preparation of commonly used dental cement. Indeed, the composition of mineral trioxide aggregate (MTA) with powder and liquid (p/l) preparation was previously dominated by 41.58% oxygen, 36.37% calcium, and 14.16% carbon. 2 3 4 By contrast, Biodentine (BD), which represents a development of MTA, has a composition with p/l preparation that is dominated by 37% calcium, 30% oxygen, and 25% carbon, while BC is a ready-to-use calcium silicate–based cement consisting of 34.81% carbon, 34.51% oxygen, 13.29% calcium, and 13.83% zirconium. 2 5 Previous research has shown that BD at a concentration of 1:5 can stimulate the odontogenic differentiation of human dental pulp stem cells (hDPSCs) better than at a concentration of 2:1, whereas there has been no equivalent research on BC, 6 7 8 although studies have shown that BC at a concentration of 1:1 increases the effect of hDPSC proliferation when compared with a concentration of 1:4. However, no studies have examined the effect of BC on the odontogenic differentiation of hDPSCs.

Odontogenic differentiation represents a determinant of the capability of pulp mesenchymal cells to regenerate the dentinal–pulp complex. 9 After the migration and proliferation of pulp mesenchymal cells occur and transforming growth factor-β1 expression is triggered, hDPSCs undergo changes in appearance to become odontoblast-like cells and structural changes to secrete specific odontogenic proteins—namely, dentin sialophosphoprotein (DSPP) and bone sialoprotein (BSP). 3 7 8 10 DSPP is a noncollagenous protein that is most commonly found in dentin. It is a regulator of dentin formation and can be used as a sign of dentin formation during the regeneration of the dentin–pulp complex. BSP is a noncollagenous protein that participates in gene coding and functions as a regulator during the formation of hard tissues such as dentin, cementum, and alveolar bone. 11 Thus, mineral deposition can provide confirmation of odontogenic differentiation. In previous research, DSPP expression was usually studied on days 7 or 14 and then confirmed via alizarin red staining on day 21 to determine if mineral deposition had occurred. 3

Therefore, the present study sought to compare the ability of BC and BD in relation to odontogenic differentiation by evaluating the DSPP and BSP expression and mineral deposition of hDPSCs. The null hypothesis was that there is no difference in odontogenic differentiation between BD and BC. 12 13 14 15 16

## Materials and Methods

This study was approved by the ethics committee of Faculty of Dentistry, Universitas Indonesia on March 28, 2023 (approval number 02/Ethical Exempted/FKGUI/2023 No. Protocol 050040123 and 09/Ethical Exempted/FKGUI/III/2023 No. Protocol 050120223). The research was conducted at the Prodia Stem Cell (ProSTEM) Laboratory in Jakarta, Indonesia. Two operators performed all the experiments.

### Premixed Calcium Silicate Cement Putty

In this research, BC was used as the premixed calcium silicate cement putty. The BC was prepared in a mold with a depth of 2 mm and a diameter of 5 mm. After setting for 48 hours, it was crushed and sterilized. Then, 3.5 mg of finely ground BC was taken, mixed with 3.5 mL of Dulbecco's modified eagle medium (DMEM; Thermo Fisher Scientific Inc., Massachusetts, United States) solution, and incubated for 24 hours at a temperature of 37°C. Next, this solution was filtered through a 0.22-µm sterile filter (ISO 10993-5). Finally, solutions with concentrations of 1:2 and 1:5 were prepared by diluting the 1:1 solution with DMEM solution using the following dilution formula: M1V1 = M2V2.

### Powder/Liquid Calcium Silicate Cement

BD, a powder/liquid calcium silicate cement, was used in this study. The BD was mixed according to the manufacturer's instructions and then placed in a mold that was 2 mm deep and 5 mm in diameter. After setting for 48 hours, the BD was crushed and sterilized. Next, 3.5 mg of finely ground BD was taken, mixed with 3.5 mL of DMEM solution, and incubated for 24 hours at a temperature of 37°C. This solution was filtered through a 0.22-µm sterile filter (ISO 10993-5). Solutions with concentrations of 1:2 and 1:5 were prepared by diluting the 1:1 solution with DMEM solution according to the following dilution formula: M1V1 = M2V2.

### hDPSC Culture

The study used hDPSCs that were obtained from nine healthy donors. These donors met the inclusion criteria of the study which included being between the ages of 18 and 25 years, having no systemic diseases, being healthy, and having no smoking or alcohol consumption habits. The hDPSCs from the third and fourth passages (P3 and P4) were isolated and cultured until reaching 80% confluence. The cells were starved for 24 hours in DMEM (Thermo Fisher Scientific Inc. ) supplemented with 1% fetal bovine serum. To create osteogenic conditions, the media were supplemented with 10 mM beta-glycerophosphate, 50 µg/mL ascorbic acid, and 100 Nm dexamethasone after 24 hours. The hDPSCs were seeded in seven different groups, as shown in Table 1 . All groups had three biological triplicates (Triplo) and ran twice during the experiment (based on ISO 10993-5).

**Table 1:** Control and experimental groups

Group	Culture media group	Consistency
BD 1	BD 1:1	hDPSCs + dilution of DMEM– BD (3.5 mg/3.5 mL)
BD 2	BD 1:2	hDPSCs + dilution of BD 1:1 (M1V1 = M2V2)
BD 3	BD 1:5	hDPSCs + dilution of BD 1:1 (M1V1 = M2V2)
BC 1	BC 1:1	hDPSCs + dilution of DMEM– BC (3.5 mg/3.5 mL)
BC 2	BC 1:2	hDPSCs + dilution of premixed BC 1:1 (M1V1 = M2V2)
BC 3	BC 1:5	hDPSCs + dilution premixed BC 1:1 (M1V1 = M2V2)
	Positive control group	hDPSCs + DMEM

Abbreviations: BC, Bio-C Repair; BD, Biodentine; DMEM, Dulbecco's modified eagle medium; hDPSCs, human dental pulp stem cells.

### DSPP and BSP Expression of hDPSCs

The hDPSCs were incubated in 96-well plates, which contained 5 × 10 ^3^ cells per well. After 7 and 14 days of incubation, the DSPP and BSP expression levels in each group were evaluated. To accomplish this, enzyme-linked immunosorbent assays were performed according to the manufacturer's protocol using a microplate reader at a wavelength of 405 nm.

### Alizarin Red Test and Quantification

Following a 21-day incubation period, both the control and experimental groups underwent alizarin red staining. Subsequently, the samples underwent microscopic examination at ×10 magnification and were photographed twice. The images were analyzed using ImageJ software (National Institutes of Health) to create descriptive profiles of the densities of the observed mineral deposition areas, which were translated into area percentages. An elevated percentage of red areas containing mineral dots indicates a greater degree of mineral deposition.

### Data Analysis

The gathered data were analyzed using a one-way analysis of variance (ANOVA), followed by Tamhane's post hoc test, to compare between the study groups. The *t* -test dependent test was also used to identify and compare differences between the study groups. All the tests were conducted at a significance level of 95% ( *p* < 0.05), and all the data were analyzed using IBM SPSS Statistics Software, version 23.0.

## Results

### Isolation and Characterization of hDPSCs

**Fig. 1:**
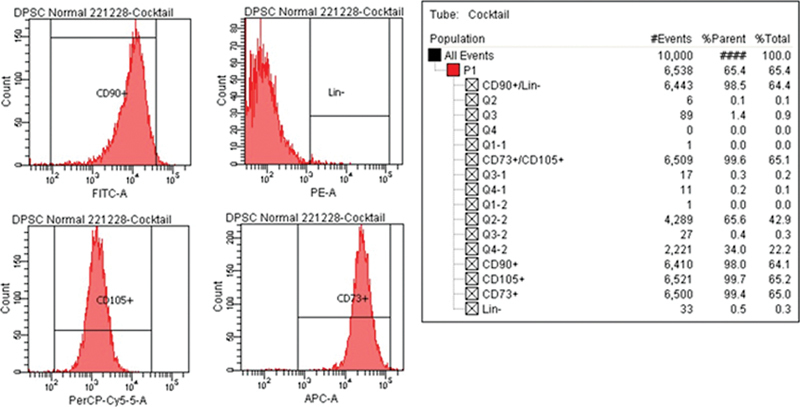
Characterization of human dental pulp stem cells (hDPSCs).

The hDPSCs were tested for their mesenchymal stem cell status using flow cytometry. The results showed that 98% of the cells had the CD 90+ marker, 99.7% had the CD 105+ marker, and 94% had the CD 73+ marker. Only 0.5% of the cells had hematopoietic markers (LinNeg), indicating that the hDPSCs had the relevant mesenchymal markers and did not have the relevant hematopoietic markers ( Fig. 1 ).

### Effect of BC on DSPP and BSP Expression in hDPSCs

**Fig. 2:**
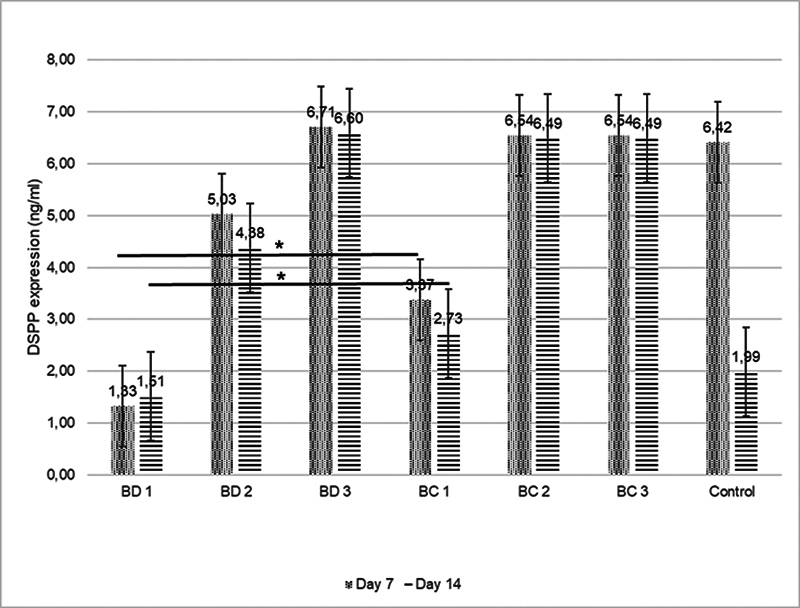
Comparison of DSPP expression between the control and experimental groups after 7 and 14 days of observation (* *p* < 0.05). BC, Bio-C Repair; BD, Biodentine; DSPP, dentin sialophosphoprotein.

**Fig. 3:**
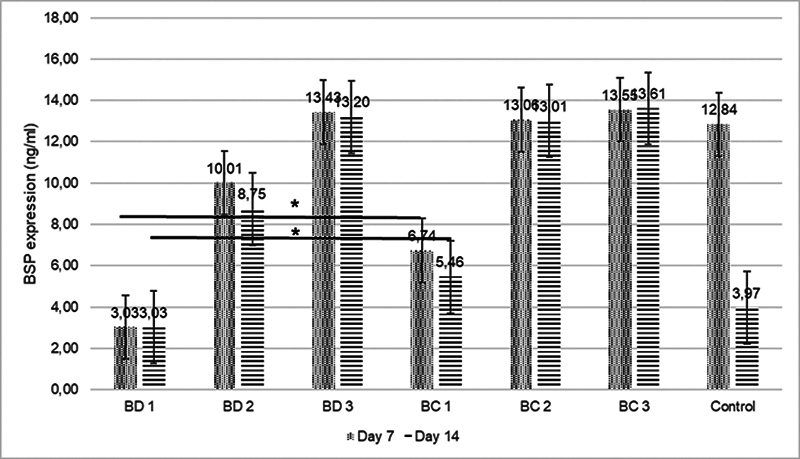
Comparison of BSP expression between the control and experimental groups after 7 and 14 days of observation (* *p* < 0.05). BC, Bio-C Repair; BD, Biodentine; BSP, bone sialoprotein.

Based on the normality test (Shapiro–Wilk, *p* ≥ 0.05), the results showed a normal distribution of the data, so parametric statistical tests were continued using one-way ANOVA with 95% confidence interval and Tamhane's post hoc test to compare each group. The one-way ANOVA demonstrated a significant difference in the DSPP and BSP expression levels in the BC 1, BC 2, and BC 3 groups when compared with the BD 1, BD 2, and BD 3 groups at 7 and 14 days of observation. Thus, changing the chemical composition of the BC influenced the DSPP and BSP expression in the hDPSCs. Fig. 2 indicates that the BC groups showed the highest level of DSPP expression at a concentration of 1:5 (BC 3) at an observation time of 7 days, while they showed the lowest at a concentration of 1:1 (BC 1) at an observation time of 7 days. On the seventh day of observation, only groups BD 2, BD 3, and BC 3 showed higher results than the positive control. Therefore, various concentrations of BC and BD can increase the DSPP expression in hDPSCs until day 14 when compared with the positive control group, except for the concentration 1:1 used in BD. In terms of the BSP expression in the hDPSCs, the same pattern is shown in Fig. 3 .

### Alizarin Red Staining and Quantification

**Fig. 4:**
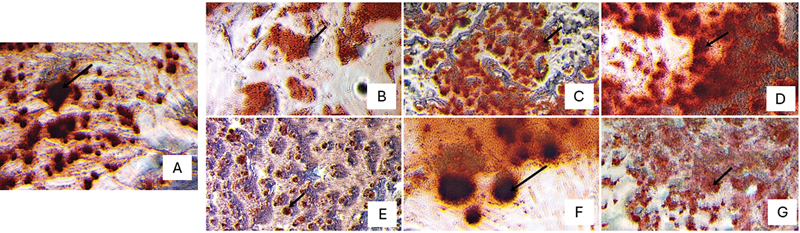
Alizarin red staining after 21 days of observation: ( **A** ) Positive control group, ( **B** ) BC 1, ( **C** ) BC 2, ( **D** ) BC 3, ( **E** ) BD 1, ( **F** ) BD 2, and ( **G** ) BD 3. Based on the alizarin red staining test after 21 days of observation, the highest levels of nodule minerals were shown in the 1:5 concentrations. BC, Bio-C Repair; BD, Biodentine.

**Fig. 5:**
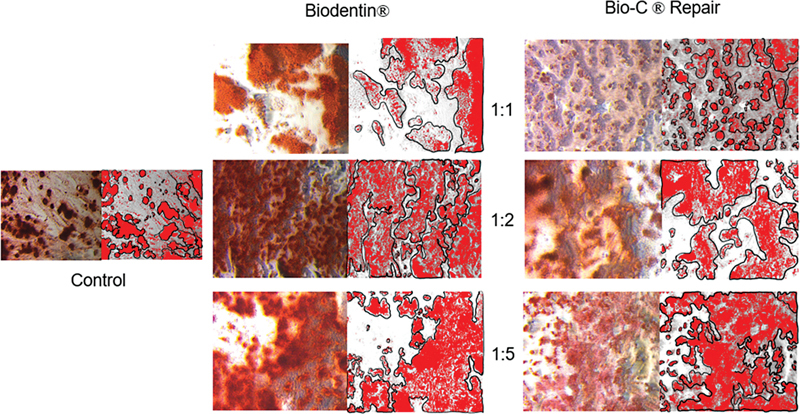
Quantification of the alizarin red staining area using ImageJ software. The calculated mineral deposition area is the red area surrounded by the black line. The percentage of the mineral deposition area in BD 1: 21.61% ± 0.78, BD 2: 33.82% ± 1.36, BD 3: 42.42% ± 0.69, BC 1: 21.50% ± 0.73, BC 2: 38.50% ± 1.31, and BC 3: 42.37% ± 1.32. BC, Bio-C Repair; BD, Biodentine.

ImageJ software was used to calculate the percentage of the mineral deposition area marked by red staining. Fig. 4 presents the results of the alizarin red staining at 21 days of observation. The percentage of the mineral deposition area found to be higher than in the control group is shown for the BD 2, BD 3, and BC 3 groups. The lowest mineral deposition area was observed in the case of BC 1, while the highest was observed for BD 3. The quantification of the mineral deposition area is shown in Fig. 5 .

## Discussion

The structure and response of the dentin–pulp complex is closely related to the activity of the odontoblasts and other cells in the pulp during their lifetime. If the pulp is healthy, it will continue to deposit tertiary dentin when an irritant is present in the tooth. If the pulp is necrotic, this process will cease. Dentine extracellular matrix is composed of proteoglycans and noncollagenous proteins. The extracellular matrix found in dentin is made up of proteoglycans and noncollagenous proteins. The noncollagenous proteins consist of small integrin-binding ligands, N-linked glycoprotein (SIBLING), which includes DSPP, dentin matrix protein 1, BSP, osteopontin, and matrix extracellular phosphoglycoprotein. SIBLING protein plays a crucial role in the mineralization of collagen fibrils and crystal growth during the conversion of predentine to dentine. This study analyzed the potential for odontogenic differentiation through the expression of DSPP and BSP.

DSPP is the most abundant noncollagenous protein found in dentin, and it is a gene coding which is the specific factor for the differentiation of hDPSCs into odontoblasts. BSP is one of the noncollagenous SIBLING proteins that serve as markers of osteogenic differentiation. 11 BSP plays a significant role as a potential nucleator of hydroxyapatite and a specific marker of both osteoblast and cementoblast differentiation. In addition, BSP is known to be an important marker of osteogenic differentiation. 6 11 17 18

BC has different physicochemical and biological properties when compared with BD, as has been discussed. These differences are due to changes in chemical composition and particle size. BC is considered a ready-to-use cement because the powder and liquid do not need to be mixed beforehand. This means that the material does not undergo hydrolysis during application. Instead, the hydrolysis process is initiated upon contact with biological fluids. This means that the pH of the cement is initially high when it comes into contact with tissue. 5 12 14 15 19

The addition of carbon elements in the form of nanotubes compensates for BC by increasing the compressive and flexural strength of the cement and reducing its porosity. Campi et al found that BC has good physical properties due to its solubility, which is less than 3%, and its volume, which is more stable after setting, compared with BD. 15 BD has a solubility of more than 3% due to its high calcium ion content, meaning that a very high level of hydroxyl ions is released after mixing and contains polymers that are water soluble in the liquid; thus, the change in the cement volume is greater. By contrast, BC has lower solubility and a more stable volume due to its low porosity and homogeneous particles. 2 20 21 22

Both types of cement used in this study feature zirconium oxide as a radio-opaquer; however, the zirconia content in BC is higher than that in BD, which means that BC exhibits greater radio-opacity than BD. Moreover, the amount of zirconia in BC has also increased because the nanoparticle size of zirconia is more stable at room temperature. Zirconium oxide has been shown to increase cell proliferation and reduce inflammation in cells when compared with bismuth oxide, which renders it more appropriate for use as a radio-opaquer in calcium silicate–based cement intended to support tissue regeneration. Thus, increasing the zirconia content of BC increases its biological properties. 2 9 10 15

Based on energy dispersive X-ray analysis, Ghilotti et al found that calcium ions comprised only 13.29% of BC but 42% of BD. 2 In the study by López-García et al, the amount of released calcium ions detected via inductively coupled plasma mass spectrometry was found to be 38.32 mg/mL in BC and 177.15 mg/mL in BD. Calcium ions play an important role in the regeneration of the dentin–pulp complex. 2 10 23

When various concentrations of premixed calcium silicate–based cement putty come into direct contact with hDPSCs, an increase in DSPP expression is observed after 7 days of exposure. This proves that premixed calcium silicate–based cement preparations can increase the potential of hDPSCs to differentiate into odontoblasts. After DSPP is secreted, it is immediately transformed into dentin sialoprotein (DSP), dentin glycoprotein (DGP), and dentin phosphoprotein by BMP-1. DGP is formed through the activity of matrix metalloproteinase-20 (MMP-20) and later processed by MMP-20 so that the N terminal forms on the DGP and the C terminal on the DSP, thereby initiating the process of dentin biomineralization. Hence, the present results showed no significant difference in DSPP expression in the hDPSCs in the premixed calcium silicate–based cement putty group at 7 and 14 days of observation. 8 24 25 26 However, the results of this study show that changes in the chemical composition of BC affect the DSPP expression in hDPSCs. The results of this study showed that the differential expression of DSPP and BSP is associated with the differential cellular survival and viability of the hDPSCs. This finding is in line with the previously published study by Ghilotti et al (2020), who also investigated the effects of three different dilutions of BD, BC, and ProRoot MTA on vital pulp therapy. However, the results of both studies indicate that none of these vital pulp materials has a cytotoxic effect on the hDPSCs. 2 Meanwhile, at concentrations of 1:2 and 1:5 and exposure times of 7 and 14 days, there was no significant difference observed between the BC and BD groups.

The study results showed a significant difference ( *p* < 0.05) in the concentration of BSP between the BC and BD groups during 7 and 14 days of observation. The highest concentration of BSP was found to be at concentration 1:5. These results are consistent with Luo et al, who observed BSP expression in hDPSCs cultured in medium supplemented with BD at a concentration of 0.2 mg/mL or 1:5 that increased until day 14. 6 No studies have shown the influence of BC on BSP expression. However, Sun et al used PMP-based calcium silicate cement (EndoSequence RRM Putty and NeoPUTTY) and observed that the BSP values increased until the third week. 27 This result is in line with the findings of this current study, which detected no significant difference in the BSP concentration between BC group with concentration of 1:2 and 1:5, as well as BD groups at different concentrations. It can be concluded that BC and BD have the same ability to induce osteogenic and odontogenic differentiation in hDPSCs, as indicated by BSP expression.

The microscopic observations in this study revealed that groups BC 1, BC 2, and BC 3 had a cleaner appearance than groups BD 1, BD 2, and BD 3. This is because BC had a smaller particle size than BD. 2 15 On day 21, alizarin red staining was performed in all the treatment groups to qualitatively illustrate the mineral deposition. Quantitative analysis was performed using ImageJ software to calculate the percentage of the mineral deposition area marked by the red staining. The results showed that more mineral deposition images were found in the premixed calcium silicate–based cement group with a concentration of 1:5. This finding is consistent with the microscopic observations on days 7 and 14, where premixed calcium silicate–based cement groups exhibited better cell viability. The higher percentage of the mineral deposition area in these groups at concentrations of 1:2 and 1:5 accords with the BSP and DSPP expression identified in this study.

## Conclusion

The results of this study indicate that BC has the same ability as BD to increase the DSPP and BSP expression and mineral deposition of hDPSCs. The concentration of BC and BD that showed the highest DSPP and BSP expression and mineral deposition was the 1:5 concentration. Based on the results of this study, it is concluded that BC has the advantage of surviving in relatively humid conditions. This information can guide clinicians in selection of bioceramic materials according to their cases.
